# Design of GAA Nanosheet Ferroelectric Area Tunneling FET and Its Significance with DC/RF Characteristics Including Linearity Analyses

**DOI:** 10.1186/s11671-022-03690-8

**Published:** 2022-05-12

**Authors:** Narasimhulu Thoti, Yiming Li

**Affiliations:** 1 Parallel Scientific Computing Laboratory, National Yang Ming Chiao Tung University, Hsinchu, 300 Taiwan; 2 Electrical Engineering and Computer Science International Graduate Program, National Yang Ming Chiao Tung University, Hsinchu, 300 Taiwan; 3 Institute of Communications Engineering, National Yang Ming Chiao Tung University, Hsinchu, 300 Taiwan; 4 Institute of Biomedical Engineering, National Yang Ming Chiao Tung University, Hsinchu, 300 Taiwan; 5 Department of Electrical and Computer Engineering, National Yang Ming Chiao Tung University, Hsinchu, 300 Taiwan

**Keywords:** Ferroelectric, Gate-all-around, Internal voltage, Linearity, Nanosheet, *n*-Epitaxy, $\hbox{Si}_{1-x}\hbox{Ge}_x$, Switching energy

## Abstract

This work reports an emerging structure of gate-all-around ferroelectric area tunneling field-effect transistor (FATFET) by considering ferroelectric and a *n*-epitaxial layer enveloped around the overlapped region of the source and channel to succeed with complete area of tunneling probability. To accomplish this, ferroelectric ($\hbox{Hf}_{0.5}\hbox{Zr}_{0.5}\hbox{O}_2$) is exploited and modeled to boost the FATFET performance through internal-voltage ($V_{{\rm int}}$) amplification. The corresponding modeling approach to estimate the ferroelectric parameters along with $V_{{\rm int}}$ calculations of the metal-ferroelectric-insulator (MFIS) option through capacitance equivalent method is addressed. Using these options the proposed device outperforms effectively in delivering superior DC and RF performance among possible options of the $\hbox{Si}_{1-x}\hbox{Ge}_x$ ferroelectric TFETs. The significance of proposed design is examined with recently reported ferroelectric TFETs. Our results show 10-time advancement on the $I_{{\rm on}}$, reduced steep or average subthreshold swing (< 25 mV/dec), frequencies higher than 150 GHz, and insignificant to linearity deviations at low bias points. Furthermore, 2-order reduction in energy efficiency is succeeded with the proposed design environment.

## Background

The inevitability of new principal devices such as tunneling field-effect transistors (TFETs) is under exploration to meet the aspects of low power consumption. TFETs are the devices that deliver low off-state current ($I_{{\rm off}}$) and subthreshold swing (SS) lower than 60 mV/dec [1]. However, based on the experimental and computational works in TFETs it is identified that achieving (1) low average or steeper SS ($\hbox{SS}_{{\rm avg}}$) instead of impressive point or minimum SS ($\hbox{SS}_{{\rm min}}$), (2) high on-state current ($I_{{\rm on}}$), and (3) low energy efficiency or switching energy (SE) are the key challenges.

Line TFETs (LTFETs) through strong vertical-gate fields have been aided with substantial tunneling probability as associated to the point TFETs [2–4]. Additionally, it is identified that the ferroelectric materials can introduce excess electric field (*E*) and polarization (*P*) than the conventional dielectrics [5, 6]. Therefore, the ferroelectric materials have become beneficial to help in amplification of tunneling probability through reduction in tunneling length ($\lambda$) by the virtue of internal voltage ($V_{{\rm int}}$). Therefore, several demonstrations (theoretical and practical) have shown that the application of ferroelectricity can advance the performance of TFETs [7, 8]. Among existing ferroelectric options, the HZO as $\hbox{Hf}_{0.5}\hbox{Zr}_{0.5}\hbox{O}_2$ has been proven to be a most appropriate option in fulfillment with the experimental approaches [9]. However, the recent demonstrations still lag behind utilization of ferroelectricity due to unfitted geometrical options of TFETs (point-tunneling) that cannot harvest a complete area of tunneling.

To address the aforementioned key challenges in TFETs, the modeled device is equipped with improved area of tunneling using scaled *n*-epitaxial layer, material options with source as $\hbox{Si}_{1-x}\hbox{Ge}_x$ (with *x* = 0.4 as the Ge fraction) and gate-dielectric as $\hbox{Hf}_{0.5}\hbox{Zr}_{0.5}\hbox{O}_2$, and the gate-all-around (GAA) nanosheet geometry. The significance of scaling line tunneling (by *n*-epitaxy and overlapped source ($L_{{\rm sov}}$)) and the ferroelectric options have been demonstrated in our recent articles [10, 11]. Here, the work is extended to detailed investigation of ferroelectric effect dependency, the device reliability by analyzing the linearity behavior, and finally the cumulative comparison with recently explored structures of ferroelectric line TFETs. Here, the ferroelectric dependency elaborates modeling of ferroelectric parameters such as $V_{{\rm int}}$ calculations; influence of remnant polarization ($P_{{\rm r}}$) and coercive fields ($E_{{\rm c}}$) on the proposed geometry.

## Methods

### Device Design and Methodology

The proposed design is processed by using TCAD simulations and in the view of emerging technology nodes (e.g., sub-3-nm) [14, 15]. A 3D device simulation platform has been quantified and validated for respective models of electron band-to-band tunneling (BTBT) and trap-assisted-tunneling (TAT) models [16, 17], along with the parameter calibrations of $\hbox{Si}_{0.6}\hbox{Ge}_{0.4}$ to achieve faithful results as similar to our earlier work [18, 19]. The initial parameters to model the several physical models are brief as follows. For example, the calculation of BTBT can be evaluated based on the tunneling mass of electron and hole, energy bandgap, and so on which are the initial parameters based on materials to evaluate additional parameters. The detailed procedure and calculations that undergo were calculated in our previous article [18]. Current article highlights the procedure to model the ferroelectric parameters, where $E_{{\rm c}}$ and $P_{{\rm r}}$ are the initial guess for analyzing the parameters of ferroelectric. The detailed procedure is illustrated in subsequent sections. The design is equipped with $\hbox{Si}_{0.6}\hbox{Ge}_{0.4}$ as source to achieve a good figure of merit in terms of $I_{{\rm on}}$ and $I_{{\rm off}}$. This is due to the fact that an added Ge content reduces the effective bandgap and tunneling mass that are beneficial for tunneling rate or $I_{{\rm on}}$. In contrast, a greater reduction of bandgap or tunneling mass is proportionate for large current during off-state regime (i.e., $I_{{\rm off}}$). Therefore, an optimum addition of Ge content is most suitable for a good figure of $I_{{\rm on}}/I_{{\rm off}}$ [19].

### Modeling of Ferroelectric Parameters

**Fig. 1:**
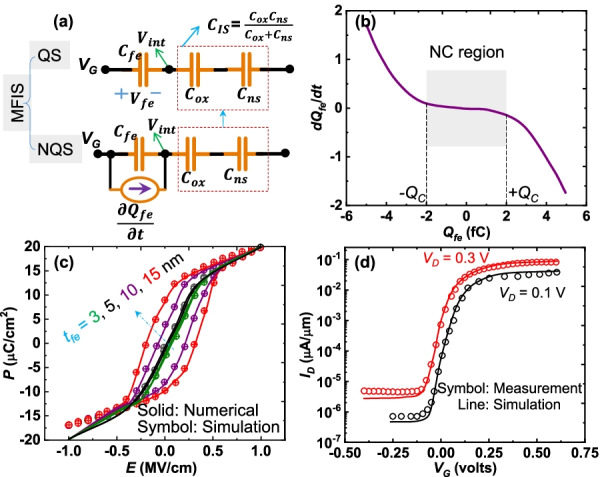
**a** Capacitance equivalent circuit with QS (bottom) and NQS (top) structure of MFIS geometry. **b** Formulated $\hbox{d}Q_{{\rm fe}}/\hbox{d}t$ with respect to $Q_{{\rm fe}}$. **c** Numerical (using L–K equation) and simulated (TCAD data) analyses of *P*–*E* relationship in the ferroelectric material at different ferroelectric thicknesses ($t_{{\rm fe}}$) [9, 12]. **d** Simulations are validated using the measurement data of a NC-TFET [6, 13]

Here, ferroelectric parameters are calibrated and plugged into simulations using Landau–Khalatnikov (L–K) approach for further strengthening of device simulations [12]. The MFIS geometry with its capacitance equivalent circuit as depicted in Fig. 1a is considered as a ferroelectric option for proposed TFETs. It is worth mentioning that several discussions have been made on quasi-static (QS) and non-quasi-static (NQS) behavior of ferroelectric capacitance ($C_{{\rm fe}}$) [20–23]. The findings are, 1) stabilization of negative capacitance (NC) at microscopic level, 2) slow switching dynamics of ferroelectricity, and 3) ambiguity between L–K (assuming $C_{{\rm fe}}$ intrinsically negative) and Miller (domain-wall propagation delay ($\tau$) as $R_{{\rm fe}}C_{{\rm fe}}$, where $R_{{\rm fe}}$ is the ferroelectric resistance) models [20]. From these findings, it has been concluded (experimentally) that the NC with QS is still valid without NQS (will be discussed further) due to slow switching dynamics of ferroelectricity at microscopic level. Hence, the L–K approach with QS based ferroelectric is modeled here.

Polarization charge in ferroelectric ($Q_{{\rm fe}})$ is [21],

1 $$Q_{{\rm fe}}=\epsilon _0\times V_{{\rm fe}}/t_{{\rm fe}}\times P\times A_{{\rm fe}}\approx P\times A_{{\rm fe}}$$

where $V_{{\rm fe}}$, $t_{{\rm fe}}$, and $A_{{\rm fe}}$ are voltage, thickness, and cross-sectional area of the ferroelectric. The rate of change of $Q_{{\rm fe}}$ is expressed as [24, 25],

2 $$\rho (\hbox{d}Q_{{\rm fe}}/\hbox{d}t)=-\hbox{d}U/\hbox{d}Q_{{\rm fe}},$$

where $\rho$ defines the frictional inertia and left-hand side of (2) represents the NQS behavior of the ferroelectric as NC. The free energy of ferroelectric (*U*) is expressed as,

3 $$U=\frac{\alpha }{2}Q_{{\rm fe}}^2+\frac{\beta }{4}Q_{{\rm fe}}^4+\frac{\gamma }{6}Q_{{\rm fe}}^6-Q_{{\rm fe}}V_{{\rm fe}}.$$

Here, $\alpha$, $\beta$, and $\gamma$ are the anisotropic constants; $V_{{\rm fe}}$ is the voltage across ferroelectric region. For simplicity, up to fourth-order polynomials (second-order phase transition) is considered. Evaluating the (2) using (3),

4 $$\rho (\hbox{d}Q_{{\rm fe}}/\hbox{d}t)=-(\alpha Q_{{\rm fe}}+\beta Q_{{\rm fe}}^3-V_{{\rm fe}}).$$

Under steady-state condition of ferroelectric ($\hbox{d}Q_{{\rm fe}}/\hbox{d}t=0$),

5 $$V_{{\rm fe}}=\alpha Q_{{\rm fe}}+\beta Q_{{\rm fe}}^3.$$

In an isolated ferroelectric region $V_{{\rm fe}} = 0$ and $Q_{{\rm fe}}=\pm Q_0$, where $Q_0$ is the initial polarization charge in the ferroelectric, implies

6 $$Q_0=\pm \sqrt{-\alpha /\beta }$$

and

7 $$\hbox{d}V_{{\rm fe}}/\hbox{d}Q_{{\rm fe}}=\alpha +3\beta Q_{{\rm fe}}^2.$$

In general, the charge in ferroelectric fluctuates from two magnitudes as $+Q_{{\rm c}}$ to $-Q_{{\rm c}}$ (Fig. 1b) at $V_{{\rm fe}} = V_{{\rm c}}$, which implies $\hbox{d}V_{{\rm fe}}/\hbox{d}Q_{{\rm fe}}=0$. Thus, $Q_{{\rm c}}$ and $V_{{\rm c}}$ are evaluated as,

8 $$Q_{{\rm c}}=\sqrt{-\alpha /3\beta }$$

and

9 $$V_{{\rm c}}= -\alpha Q_{{\rm c}}-\beta Q_{{\rm c}}^3$$

10 $$= -\left( \sqrt{\frac{-\alpha }{3\beta }}\right) \left( \alpha +\beta \left( \sqrt{\frac{-\alpha }{3\beta }}\right) ^2\right)$$

11 $$= -\left( \sqrt{\frac{-\alpha }{3\beta }}\right) \left( \frac{2\alpha }{3}\right) =Q_0\left( -\frac{2\alpha }{3\sqrt{3}}\right) .$$

By employing (6) in (11), the $\alpha$ and $\beta$ can be derived as,

12 $$\alpha =-3\sqrt{3}V_{{\rm c}}/(2Q_0)$$

and

13 $$\beta =+3\sqrt{3}V_{{\rm c}}/(2Q_0^3).$$

External electric field ($E_{{\rm ext}}$) in the ferroelectric depends on the applied voltage (*V*), i.e.,

14 $$E_{{\rm ext}}=V/t_{{\rm fe}}.$$

Hence, $V_{{\rm c}}$ and $Q_0$ are functions of the coercive field ($E_{{\rm c}}\approx V_{{\rm c}}/t_{{\rm fe}}$) and *P* (see (1)), respectively. In addition, the factor of remnant polarization ($P_{{\rm r}}$) is derived from *P*, i.e.,

15 $$P=\left( \epsilon _0\chi +\frac{P_{{\rm r}}}{E_{{\rm c}}}\right) E_{{\rm ext}},$$

where $\chi$ represents the electric susceptibility of the ferroelectric and $E_{{\rm c}}\le E_{{\rm ext}}\le E_{{\rm c}}$. The $E_{{\rm c}}$ and $P_{{\rm r}}$ are measured experimentally as $\approx$ 1 MV/cm and 1–20 μC/cm^2^ by properly controlling the ferroelectricity in $\hbox{Hf}_{1-x}\hbox{Zr}_{{x}}\hbox{O}_2$ [9]. Therefore, using expressions (12)–(15), the evaluated $\alpha$ and $\beta$ at $P_{{\rm r}}=10$ μC/cm^2^ and $E_{{\rm c}} = 1$ MV/cm are of $-1.299\times 10^{11}$ cm/F and $6.4952\times 10^{20}$ cm$^5$/FC^2^, respectively. Recalling (5), the $V_{{\rm fe}}$ can be identified to be a beneficial factor for the enhancement of $V_{{\rm int}}$ to achieve high tunneling probability in TFETs. The numerically solved data (above expressions) and the extracted data from TCAD simulations are depicted in Fig. 1c. This signifies the simulations are properly tuned according to the standard expressions. In addition, simulations are also calibrated with measurement (or experimental [6]) data and are observed as consistent, which is depicted in Fig. 1d [13].

### Modeling of $V_{{\rm int}}$

The formation of $V_{{\rm int}}$ for the MFIS (Fig. 1a) can be evaluated through a voltage-divider rule for the series connected lumped elements of capacitance (since distributed charge on the oxide and ferroelectric is the product of capacitance and potential). Here, $V_{{\rm int}}$ for both the QS and NQS is evaluated as follows.

Based on Fig. 1a (top) i.e., QS, the $V_{{\rm int}}$ is expressed as,

16 $$V_{{\rm int}}=\frac{[1/C_{{\rm IS}}+1/C_{{\rm fe}}]^{-1}}{C_{{\rm IS}}}V_{{\rm G}}$$

17 $$=V_{{\rm G}}\left( \frac{C_{{\rm fe}}}{C_{{\rm fe}}+C_{{\rm IS}}}\right) ,$$

where $C_{{\rm IS}}$ and $C_{{\rm fe}}$ are the capacitance across insulator-semiconductor and the ferroelectric regions, respectively.

The $V_{{\rm int}}$ of NQS is evaluated by applying the current rule in Fig. 1a (bottom),

18 $$C_{{\rm IS}}\frac{\hbox{d}V_{{\rm int}}}{\hbox{d}t}+C_{{\rm fe}}\frac{\hbox{d}(V_{{\rm int}}-V_\mathrm{G})}{\hbox{d}t}=C_{{\rm fe}}\frac{\hbox{d}Q_{{\rm fe}}}{\hbox{d}t}.$$

Note that the applied and internal voltages ($V_\mathrm{G}$ and $V_{{\rm int}}$) are assumed to be variation with time. Further (18) simplifies as,

19 $$\frac{\hbox{d}V_{{\rm int}}}{\hbox{d}t}(C_{{\rm fe}}+C_{{\rm IS}})= C_{{\rm fe}}\frac{\hbox{d}V_{{\rm G}}}{\hbox{d}t}+C_{{\rm fe}}\frac{\hbox{d}Q_{{\rm fe}}}{\hbox{d}t}$$

20 $$\frac{\hbox{d}V_{{\rm int}}}{\hbox{d}t}= \frac{C_{{\rm fe}}}{(C_{{\rm fe}}+C_{{\rm IS}})}\frac{\hbox{d}V_{{\rm G}}}{\hbox{d}t}+\frac{C_{{\rm fe}}}{(C_{{\rm fe}}+C_{{\rm IS}})}\frac{\hbox{d}Q_{{\rm fe}}}{\hbox{d}t}$$

Switching the factors of $\hbox{d}V_{{\rm int}}/\hbox{d}t$, $\hbox{d}V_{{\rm G}}/\hbox{d}t$ as $\Delta V_{{\rm int}}$ and $\Delta V_\mathrm{G}$, respectively, then

21 $$\frac{\Delta V_{{\rm int}}}{\Delta V_{{\rm G}}}=\frac{C_{{\rm fe}}}{(C_{{\rm fe}}+C_{{\rm IS}})}+\frac{\hbox{d}Q_{{\rm fe}}/\hbox{d}t}{\Delta V_{{\rm G}}}\frac{1}{(C_{{\rm fe}}+C_{{\rm IS}})}.$$

The expressions (17) and (21) signify the $V_{{\rm int}}$ or $\Delta V_{{\rm int}}$ calculations for QS and NQS; the $V_{{\rm int}}$ of QS can be visualized in (21) at $\partial Q_{{\rm fe}}/\partial t\rightarrow 0$. As a result, higher $V_{{\rm int}}$ implies lower $V_{{\rm G}}$ requirements, meaning that operating at low voltage is beneficial to the device.

According to NC-FET studies, the gate-oxide ($t_{{\rm ox}}$) has to be thinner to utilize the benefits of ferroelectricity and hysteresis free operation [27]. The thinner $t_{{\rm ox}}$ plays the role of $\lambda$ reduction through its resultant low effective oxide thickness ($\hbox{EOT}\,\rightarrow \,t_{{\rm ox}}$) [28], which is beneficial for improved TFETs performance. In addition, the use of low nanosheet geometrical thickness ($t_{{\rm ns}}$) can further shrink the $\lambda$, based on [28]

22 $$\lambda _{{\rm GAA}}=\sqrt{\frac{2\epsilon _{{\rm ns}}t_{{\rm ns}}^2ln(1+2t_{{\rm ox}}/t_{{\rm ns}})+\epsilon _{{\rm ox}}t_{{\rm ns}}^2}{16\epsilon _{{\rm ox}}}}.$$

Here, $\epsilon _{{\rm ns}}$ and $\epsilon _{{\rm ox}}$ are the permittivity of nanosheet and gate-oxide. Hence, the low $t_{{\rm ox}}$ or EOT in association with low $t_{{\rm ns}}$ can afford enough source to have reasonably high electron BTBT at the tunneling junction by dropping $\lambda$ and amplifying $V_{{\rm int}}$.

### Design of ATFET and FATFET

**Fig. 2:**
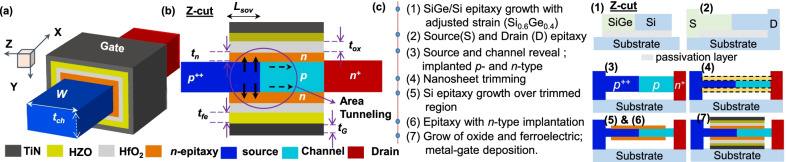
**a** The proposed structure of GAA nanosheet FATFET and **b** its 2D view along the Z-cut, showing device specifications, regions, and location of area tunneling. An *n*-epitaxial region surrounds the overlapped source ($L_{{\rm sov}}$) and channel regions to achieve complete area of tunneling. **c** The simplified processing mechanisms for the proposed geometry based on recent inventions

The demonstration of the GAA nanosheet structure of FATFET is depicted in Fig. 2. Figure 2a illustrates a novel design by stacking *n*-epitaxial layer over the channel and source overlapped regions ($L_{{\rm sov}}=5$ nm) to improve the factor of vertical tunneling. The complete (area of) tunneling along the source ($p^{++}$)-*n* and $p^{++}$-channel (*p*) can be recognized (arrows) in Fig. 2b. In addition, the drain region expansion is ignored here compared to our previously reported structure [28] because we identified that the drain expansion has the least significance on the tunneling rate and this ignorance will be beneficial for reduction in device complexity at fabrication environment.

A simplified version of Fig. 2c depicts the processing steps necessary to build the proposed device architecture. The process begins with the epitaxy of SiGe/Si on top of a passivation layer over a substrate. This passivation layer can be utilized to generate multiple nanosheets and also for GAA employment at the end. In continuation, the source and drain epitaxy can be grown on top of the substrate. The process is continued for source–channel revealing through elimination of the passivation layer, subsequently *p*- and *n*-type implantations for source(S), channel, and drain (D) regions. The nanosheet must then be trimmed to replace with epitaxy (*n*) growth. Recent demonstrations have shown growth of 4 nm thick nanosheets with GAA nanosheet Si/SiGe epitaxy using 2 nm trimming [29]. Further, the trimmed nanosheet is substituted with epitaxy layer growth and trailed by selective doping of *n*-type implantation aimed at epitaxy region. The advanced processing techniques like metalorganic vapor-phase epitaxy (MOCVD) [30], neutral beam etching (NBE) can be employed for growth and etching mechanisms [31]. At last, the gate-oxide and ferroelectric layer can be deposited by atomic layer deposition (ALD) and or pulsed layer deposition (PLD) [32], followed by TiN deposition for metal formation. The device specifications and materials used are listed in Table 1.

**Table 1:** Device design specifications and materials

Parameter	Material	Value
Gate length ($L_{{\rm G}}$)	Si	15 nm
Source/drain length	$\hbox{Si}_{0.6}\hbox{Ge}_{0.4}$/Si	15 nm
Source overlap length ($L_{{\rm sov}}$)	Si	5 nm
Channel thickness ($t_{{\rm ch}}$)	Silicon	5 nm
Epitaxy thickness ($t_\mathrm{n}$)	Si	2 nm
Effective channel thickness ($t_\mathrm{n}+t_{{\rm ch}}+t_\mathrm{n}$)	Si	9 nm
Channel width (*W*)	Silicon	10 nm
Oxide thickness ($t_{{\rm ox}}$)	$\hbox{HfO}_{{2}}$	3 nm
ferroelectric thickness ($t_{{\rm fe}}$)	$\hbox{Hf}_{0.5}\hbox{Zr}_{0.5}\hbox{O}_2$	3 nm
Source doping concentration ($p^{++}$)	Boron	$5\times 10^{20}$
Channel doping concentration (*p*)	Boron	$1\times 10^{16}$
Drain doping concentration ($n^{+}$)	Arsenic	$1\times 10^{19}$
Epitaxy doping concentration (*n*)	Arsenic	$5\times 10^{18}$
Gate-metal thickness ($t_{{\rm G}}$)	TiN	3 nm
Gate-electrode work-function	TiN	4.36 eV

**Fig. 3:**
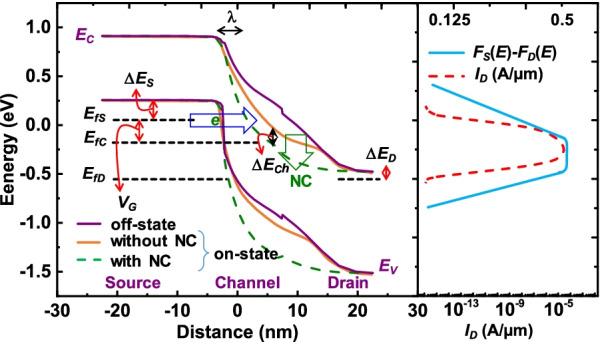
The energy band diagram of FATFET with Fermi levels (evaluates the performance of tunneling), NC effect (induce of $V_{{\rm fe}}$), extracted current density or $I_\mathrm{D}$, and difference between Fermi levels of source ($F_{{\rm S}}(\mathrm{E})$) and drain ($F_{{\rm D}}(\mathrm{E})$). Induced $V_{{\rm fe}}$ amplifies $V_{{\rm int}}$ and reduces the $\lambda$; proportionally steep SS and high $I_{{\rm D}}$ are achieved

The principle mechanism of ATFET (without ferroelectric) and FATFET works on both vertical or line and horizontal or lateral tunneling approaches (see Fig. 2b with vertical and horizontal arrows). Point tunneling is the other approach that leads to lateral tunneling with gate-edge field (hence, point tunneling) [4]. Notably, the concept of line tunneling is a leading mechanism than the point tunneling [33]. However, the line tunneling requires higher biasing voltages to exhibit its significance than point tunneling. Therefore, the ferroelectric material can be able to solve this issue by inducing an additional $V_{{\rm int}}$ to reduce the bias level of line tunneling, i.e., by FATFET. The total external voltage as the applied gate-voltage ($V_\mathrm{G}$) and $V_{{\rm int}}$ at reasonable drain-bias ($V_\mathrm{D}$) will make enough band bending, resulting in sharp reduction of $\lambda$. Besides, the scaled *n*-epitaxial region and $L_{{\rm sov}}$ provides enough room to accommodate a large area of BTBT. The relevant information is depicted in Fig. 3, showing the energy band profile during off- ($V_\mathrm{G}=0$ V, $V_\mathrm{D}=0.5$ V) and on-states ($V_\mathrm{G}=V_\mathrm{D}=0.5$ V) along source–channel–drain regions of with and without NC (by ferroelectric) devices. The effect of source, channel, and drain Fermi levels ($E_{{\rm fs}}, E_{{\rm fc}}$, and $E_{{\rm fd}}$) along with their reductions in energy levels: $\Delta E_\mathrm{s}, \Delta E_{{\rm ch}}$ and $\Delta E_\mathrm{d}$, are evaluated and depicted. Here, the slope of current density (Fig. 3(right)) can be reduced through large $V_{{\rm int}}$; ultimately, it helps to accomplish low $\hbox{SS}_{{\rm avg}}$. Thus, the grouping of ferroelectric film ($\hbox{Hf}_{0.5}\hbox{Zr}_{0.5}\hbox{O}_2$), $\hbox{Si}_{0.6}\hbox{Ge}_{0.4}$ as source, and selectivity of *n*-epitaxial layer with nanosheet geometry options would consequently improve characteristics of FATFET.

## Results and Discussion

The significance of FATFET with respect to ATFET in terms of line tunneling rate, DC-RF, and linearity analyses is explored here. The discussions are further extended to cumulative comparison of the DC characteristics and SE evaluations of proposed and the explored structures.

### Energy Band Calculations

The energy band structure of the proposed FATFET is extracted and analyzed (Fig. 3) based on the energy and built-in potential calculations, as explained below. The terms represented in the energy band diagram (Fig. 3), i.e., $\Delta E_\mathrm{s}$, $\Delta E_{{\rm ch}}$ and $\Delta E_{{\rm d}}$, can be defined with $\Delta E_{{\rm s}}=E_{{\rm VBmax}}-E_\mathrm{f}$ of the source, $\Delta E_{{\rm ch}}=E_\mathrm{f}-E_{{\rm CBmin}}$ of the channel, and $\Delta E_{{\rm d}}=E_\mathrm{f}-E_{{\rm CBmin}}$ of the drain, respectively. Here, $E_{{\rm CBmin}}$ and $E_{{\rm VBmax}}$ are the conduction band minimum and valence band maximum. The calculated $\Delta E_{{\rm s}}$, $\Delta E_{{\rm ch}}$, and $\Delta E_{{\rm d}}$ (difference in Fermi level) are $-0.096$, 0.195, and $-0.0304$ eV for FATFET. Since the source is strained with that of $\hbox{Si}_{1-x}\hbox{Ge}_x$ with $x = 0.4$, the effective mass of density of states ($m_\mathrm{V}$) in valence band is calculated via linear approximation, as [34]

23 $$m_\mathrm{V}(x)=(0.81-0.47x)m_0,$$

where $m_0$ is the electron mass in free space. Consequently, the source–channel and source–drain built-in potentials $\phi _{{\rm sc}}$ and $\phi _{{\rm sd}}$ can be expressed as

24 $$\phi _{{\rm sc}}=-\Delta E_{{\rm s}}-\Delta E_{{\rm c}}-E_\mathrm{g},$$

and

25 $$\phi _{{\rm sd}}=-\Delta E_{{\rm s}}-\Delta E_{{\rm d}}-E_\mathrm{g}.$$

The calculated built-in potentials in the proposed structure (FATFET) are of $\phi _{{\rm sc}}=-0.719$ eV and $\phi _{{\rm sd}}=-1.1364$ eV. Based on these calibrations it is clear that the higher $V_{{\rm int}}$ makes provision for deeper band bending and thus higher electron BTBT. Therefore, the current density increases with respect to the source-drain Fermi level difference ($F_{{\rm S}}(\mathrm{E})-F_{{\rm D}}(\mathrm{E})$) as depicted in Fig. 3 (right).

### Role of Ferroelectricity in FATFET Compared with ATFET

**Fig. 4:**
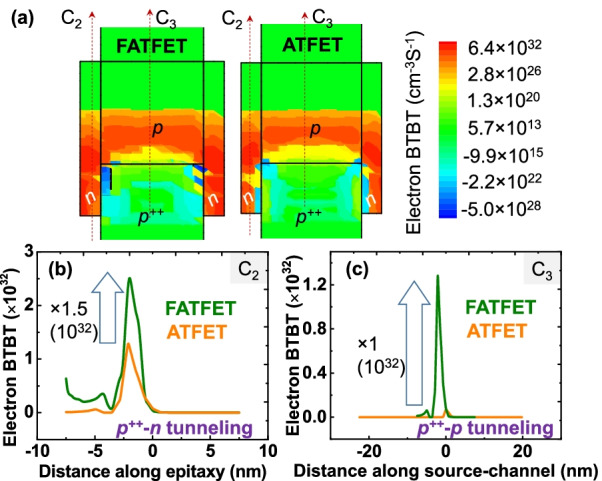
**a** Electron BTBT profile of devices FATFET and ATFET with high tunneling in FATFET (closer to $p^{++}$-*n*-*p* junctions). Plots of (**b**) vertical tunneling ($p^{++}$–*p*) and **c** lateral tunneling along the cut-lines $\hbox{C}_2$ and $\hbox{C}_3$ in the explored devices. Due to gradual decrease in tunneling length (as per Fig. 3), FATFET exhibits stronger vertical and lateral tunneling than ATFET

Due to strong vertical-fields with the benefits of high *P* and *E* through ferroelectric, the FATFET is able to attain higher electron BTBT than the ATFET, as depicted in Fig. 4. More precisely, excess BTBT rate at position $p^{++}-n$ (vertical tunneling) and $p^{++}-p$ (lateral tunneling) are observed from Fig. 4b, c through the cut-lines $\hbox{C}_2$ and $\hbox{C}_3$. It is clear that the FATFET has higher vertical (1.5 times) and lateral tunneling (1-order) rates.

**Fig. 5:**
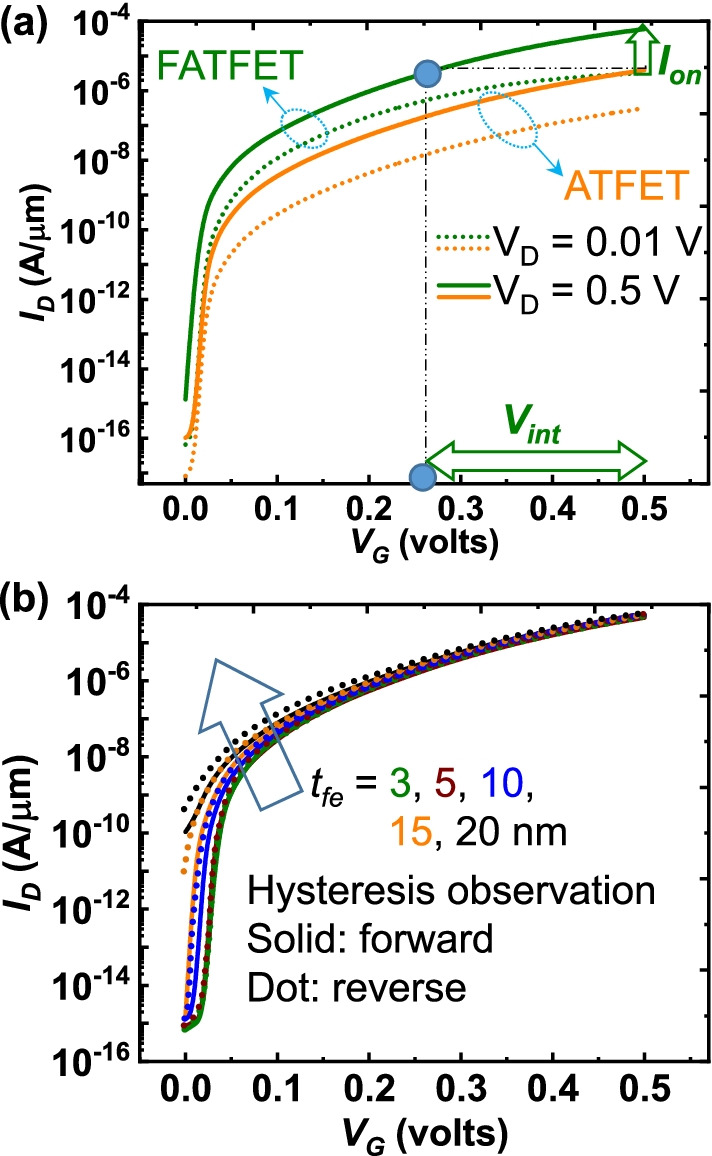
**a** Collective comparison of the FATFET and ATFETs at different drain bias points ($V_{{\rm D}} = 0.01$ and 0.5 V). A voltage gain of $\approx$ 0.2 V as $V_{{\rm int}}$ is achieved in FATFET than the ATFET that amplifies $I_{{\rm on}}$. **b** Hysteresis observation in FATFET through scaled $t_{{\rm fe}}$ during forward (solid) and reverse gate-bias sweep (dotted). Higher the $t_{{\rm fe}}$ larger will be the hysteresis

The comparative $I_{{\rm D}}$–$V_{{\rm G}}$ characteristics of ATFET and FATFETs are depicted in Fig. 5a at multiple bias points ($V_{{\rm D}} = 0.01$ and 0.5 V). With the aforementioned benefits of amplification in $V_{{\rm int}}$, reduction in $\lambda$, and tunneling rate enhancements; the FATFET achieves with high $I_{{\rm on}}$ having benefited $\approx$ 0.2 V of $V_{{\rm int}}$. The hysteresis operation is also shown by varying the $t_{{\rm fe}}$ from 3 to 20 nm. It is evident that the reduction in $t_{{\rm fe}}$ below 5*nm* suppresses the hysteresis [13, 27]. Whereas, the $I_{{\rm on}}$ affects marginally in TFETs due to its dependency on EOT. Though the $V_{{\rm fe}}$ increases proportionally with $t_{{\rm fe}}$, this will not be beneficial for TFETs to deliver high-$I_{{\rm on}}$. Since $\lambda$ enlarges for an increased $t_{{\rm fe}}$ due to high EOT (see (22)) [28]. Hence it is affordable to use low $t_{{\rm fe}}$ that will be beneficial not only the high-$I_{{\rm on}}$ (especially in TFETs) but also non-hysteresis operation.

### Significance of $P_{{\rm r}}$ and $E_{{\rm c}}$ in FATFET

**Fig. 6:**
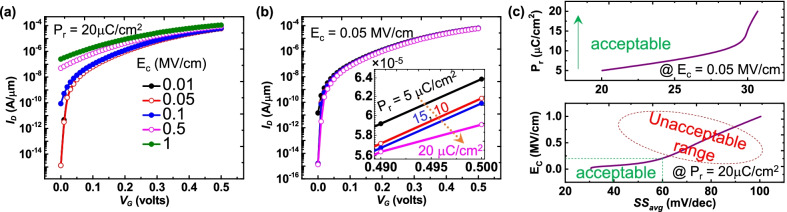
FATFET performance under fixed **a** $P_{{\rm r}}=20$ μC/cm^2^ and **b** $E_{{\rm c}}=0.05$ MV/cm. A high impact on gate leakage and tunneling through a high $E_{{\rm c}}$, resulting in high $I_{{\rm off}}$ and $I_{{\rm on}}$. **c** The significance and appropriate choice of $P_{{\rm r}}$ and $E_{{\rm c}}$ estimation from the perspective of $\hbox{SS}_{{\rm avg}}$. Similar to plot (**a**), $\hbox{SS}_{{\rm avg}}$ deteriorates at high $E_{{\rm c}}$, whereas $P_{{\rm r}}$ is insignificant

The ferroelectric parameters of $P_{{\rm r}}$ and $E_{{\rm c}}$ are crucial in estimating the device behavior for the optimized ferroelectric option. Hence, the significance of $P_{{\rm r}}$ and $E_{{\rm c}}$ is analyzed in terms of $I_{{\rm D}}$–$V_{{\rm G}}$ and SS, which are depicted in Fig. 6. The results are consistent to recent demonstrations [7], i.e., the variation in $E_{{\rm c}}$ replicates stronger influence than $P_{{\rm r}}$. Due to the fact that the tunneling is more pronounced to $E_{{\rm c}}$ and which is sensitive in vertical TFET geometries. The same can be viewed in Fig. 6a that the $>0.1$ MV/cm of $E_{{\rm c}}$ is sensitive to $I_{{\rm off}}$. Figure 6b reveals the marginal variation in performance ($I_{{\rm on}}$) at fixed $E_{{\rm c}}$ but eventually $I_{{\rm off}}$ becomes sensitive at very high $P_{{\rm r}}$. It is because $Q_{{\rm fe}}$ increases at higher $P_{{\rm r}}$ and is thus responsible for decrease in tunneling window (Fig. 3). Nevertheless, the significance of $P_{{\rm r}}$ is minor as compared to $E_{{\rm c}}$ by the view of less variation in energy level across the tunneling window. Hence, from the perspective of $\hbox{SS}_{{\rm avg}}$ and $I_{{\rm off}}$, it can be concluded that the acceptable range of $P_{{\rm r}}$ and $E_{{\rm c}}$ for an optimum ferroelectric utilization is below 30 μC/cm^2^ and 0.25 MV/cm (Fig. 6c).

### Significance of Interface Defects in FATFET

**Fig. 7:**
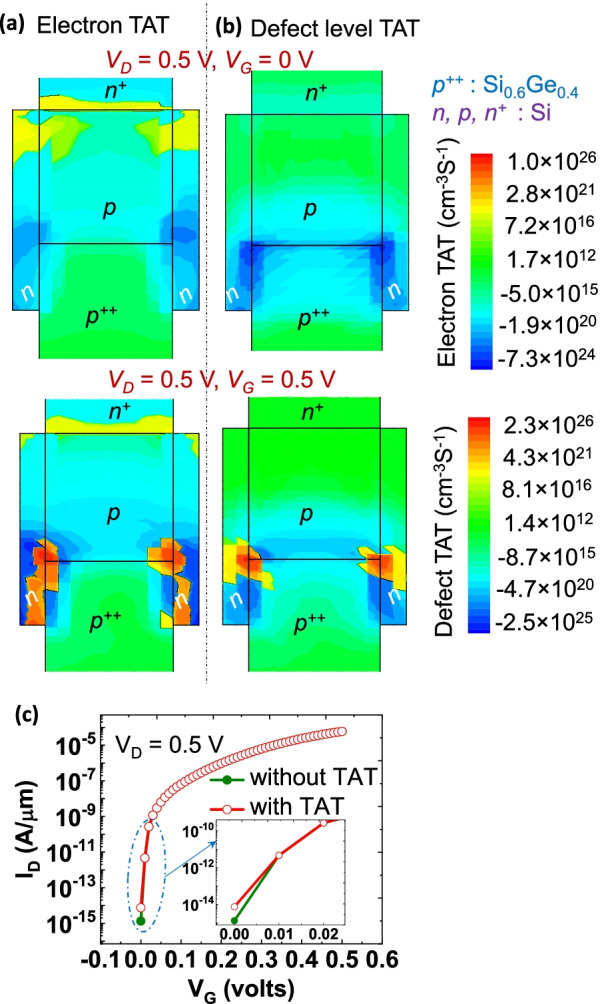
The TAT of FATFETs in both the off-state ($V_{{\rm D}}=0.5\,\hbox{V}$, $V_{{\rm G}}=0\,\hbox{V}$) and on-state ($V_{{\rm D}}=0.5\,\hbox{V}$, $V_{{\rm G}}=0.5\,\hbox{V}$) states, resulting from **a** position-dependent electron TAT and **b** defect level TAT, respectively. Position dependent electron TAT can be identified along the $p^{++}$-*n* and *p*-*n* junctions, while defect level TAT at the Si/$\hbox{Si}_{0.6}\hbox{Ge}_{0.4}$ interfaces. **c** Impact of TAT on $I_{{\rm D}}$–$V_{{\rm G}}$ characteristics; inset shows less than 1-order high $I_{{\rm off}}$ due to overall contribution of TAT

The interface and/or extended defects which can cause carrier scattering, as well as traps that are degradation factors of the transistors are explored here. It means that phonons experience scattering at interfaces and contribute to net recombination rate or TAT by which degradation of TFETs performance is observed. The influence of net recombination rate or TAT is captured by dynamic nonlocal path TAT (by Hurkx) and discussed here [16]. The model not only captures the position dependent electron and hole TAT but also the defect level TAT through phonon assisted tunneling processes. This model dynamically creates a tunneling path based on the energy profile for both aforementioned TAT’s. Here, the defect level can be identified along the tunneling path direction ($0\le X \le L$), where ’*X*’ being the tunneling path direction (similar to electron tunneling direction shown in Fig. 3), ’0’ and ’*L*’ are the starting and ending positions of tunneling path lengths. The electron and hole occupation probabilities at the defect level can be determined by balancing the net electron and hole capture rate. The simulated profile of electron TAT (i.e., position dependent) and the defect level TAT of FATFET are depicted in Fig. 7 at multiple bias points. The distribution of electron TAT can be observed along the $p^{++}$-*n* and *p*-*n* junctions, whereas defect level TAT is seen at the Si/$\hbox{Si}_{0.6}\hbox{Ge}_{0.4}$ interfaces from Fig. 7a, b. In addition, the low bias or off-state ($V_{{\rm D}}=0.5$ V, $V_{{\rm G}}=0$ V) is less effective than at high bias point ($V_{{\rm D}}=0.5\,\mathrm{V}=V_{{\rm G}}=0.5$ V). The electron TAT is more significant than defect level TAT even though the magnitude of defect level TAT is slightly higher. Nevertheless, the overall contribution of TAT on the proposed device is minor, therefore the effect of $I_{{\rm off}}$ current is less than 1-order higher as shown in Fig. 7c.

### RF and Linearity Metrics of FATFETs

**Fig. 8:**
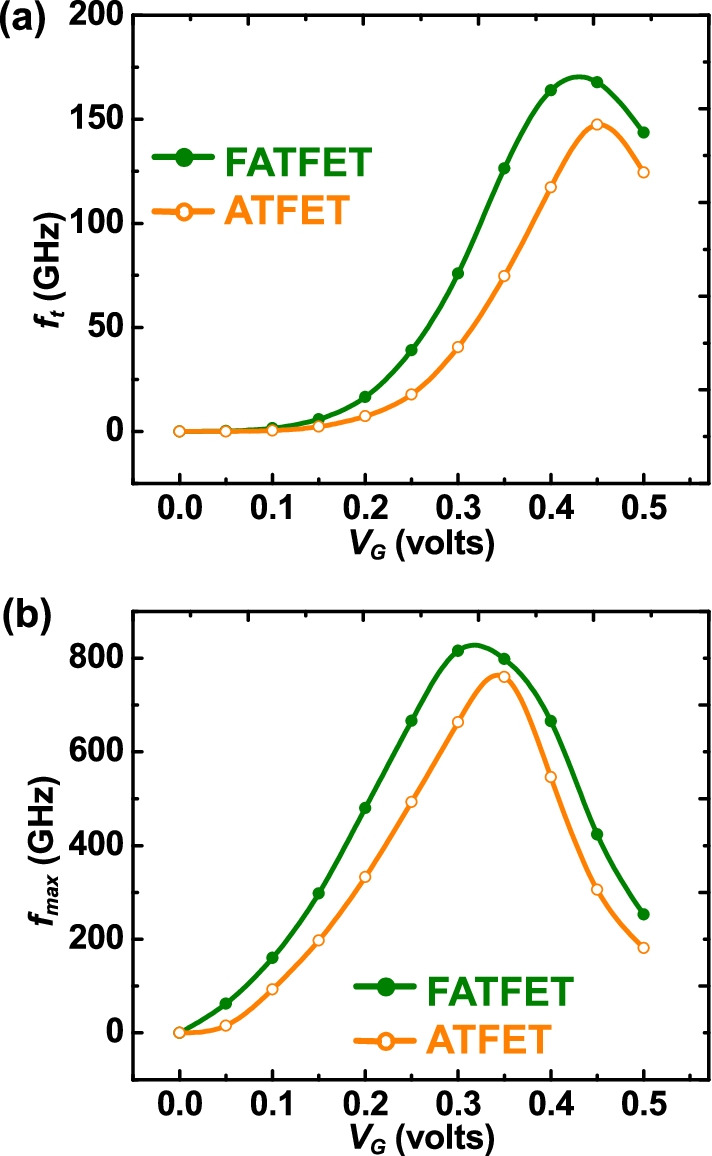
RF comparison of FATFET and ATFET in terms of **a** $f_{{\rm t}}$ and **b** $f_{{\rm max}}$. The benefit of $\approx$ 0.1 V can be achieved from FATFET at the targeted maximum frequency range of ATFET

**Fig. 9:**
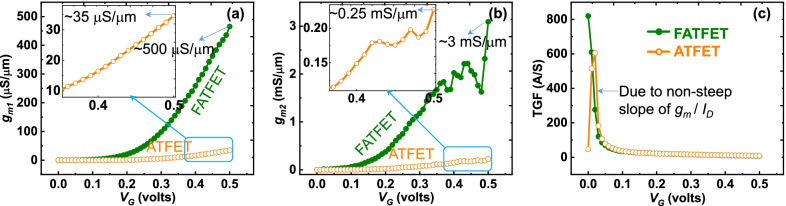
Linearity behavior with **a** $g_{m1}$, **b** $g_{m2}$, and **c** TGF of FATFET and ATFET, respectively. Compared to ATFET, FATFET has a higher transconductance and less distortion

The frequency response of FATFET and ATFET is analyzed in terms of unity-gain cutoff frequency ($f_{{\rm t}}$) and maximum oscillation frequency [35], respectively. It is to be noticed from Fig. 8 that the frequency terms are higher in FATFET than the ATFET because of its high drive current capability (i.e., $I_{{\rm on}}$) as discussed before. A substantial benefit in terms of $V_{{\rm G}}$ requirement can be noticed in FATFET compared to ATFET targeted with maximum frequency achievement. The linearity test is crucial to signify the amount of power wastage by the device due to non-linearity behavior. A non-linear device could switch the biasing point and cause reduction in gain and frequency terms. Hence, the transconductance should be high enough and low distortion oriented at the operating point (active or depletion region) of TFETs. The transconductance coefficients can be evaluated as,

26 $$g_{m_n}=\frac{\hbox{d}^nI_{{\rm D}}}{\hbox{d}V_{{\rm G}}^n},$$

where *n* = 1, 2, 3, .... For the linearity test, higher-order transconductance coefficients such as $g_{m_1}$ and $g_{m_2}$ and transconductance generation factor (TGF) are considered [36, 37]. Figure 9a depicts the $g_{m1}$ of FATFET and ATFET, stating the transconductance continuum with $V_{{\rm G}}$ without harmonic distortion in FATFET than ATFET. In addition, the FATFET is achieved with $\ge$ 10-time improvement in $g_{m_1}$ than the ATFET, whereas, higher-order $g_{m_2}$ as depicted in Fig. 9b has two or more harmonic distortions both in FATFET and ATFET, respectively. This signifies that the device is non-linear and should be taken care of. Nevertheless, the device still operates linearly during the active region (0.2–0.3 V) or below the gate-overdrive voltage ($V_{{\rm G}}-V_{{\rm t}}$) point, where $V_{{\rm t}}$ is the threshold voltage. TGF as shown in Fig. 9c shows that the FATFET has better linearity compared with ATFET. The non-linear behavior of TGF in ATFET is due to non-steep swing of $I_{{\rm D}}$–$V_{{\rm G}}$ behavior as per Fig. 5a compared with FATFET. From these analyses, it is understood that the FATFET has better linearity improvement compared with ATFET; however, more analyses are needed to address other linearity factors that will be considered in the future work.

### Significance of Proposed FATFET

**Fig. 10:**
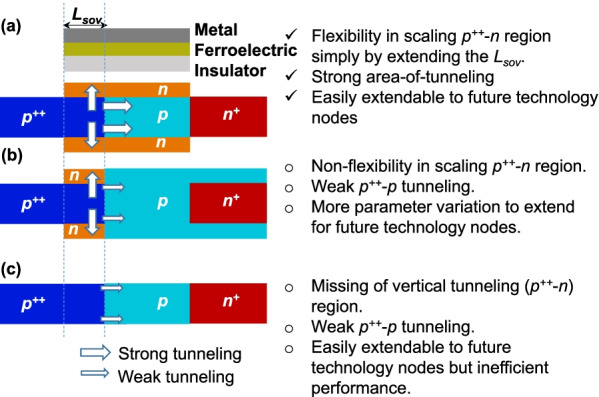
Comparative advantages of **a** proposed FATFET over **b** and **c** earlier reported structures in terms of tunneling rate and device flexibility for future technology nodes

**Fig. 11:**
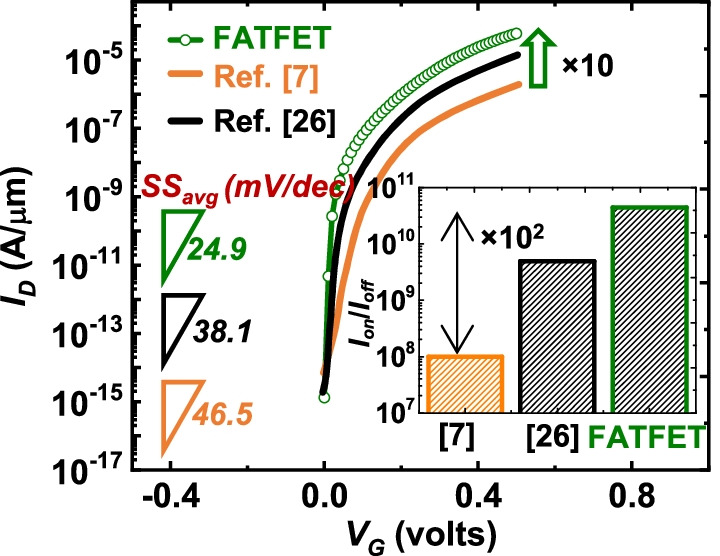
$I_{{\rm D}}$–$V_{{\rm G}}$, $\hbox{SS}_{{\rm avg}}$, and $I_{{\rm on}}/I_{{\rm off}}$ (inset) comparison with recently reported structures by re-modulating with similar specifications and material options as proposed geometry [7, 26]. The key advantages are 10-time improvement in $I_{{\rm on}}$, 2-order improvement in $I_{{\rm on}}/I_{{\rm off}}$, and improved $\hbox{SS}_{{\rm avg}}$ than the reported options

The significance of proposed geometry (FATFET) is addressed through cumulative comparison of recently reported NC-TFETs [7, 26]. Though the reported structures are with planar and dissimilar materials options, here we have re-implemented the structures ( [7, 26]) with nanosheet and $\hbox{Si}_{0.6}\hbox{Ge}_{0.4}$ as source. The geometrical comparisons are listed in Fig. 10 with merits and demerits of each (right of Fig. 10). That is, the proposed geometry (Fig. 10a) has the flexibility in scaling the tunneling junction ($p^{++}$-*n*) by simply varying the $L_{{\rm sov}}$. This resembles the advantage of area tunneling feature can easily adjusted even for future technology nodes, whereas Fig. 10a and b (the reported structures) is restricted with weak $p^{++}$–*p* tunneling. Furthermore, the explored structure in Fig. 10b is not flexible in scaling $p^{++}$-*n* junction for future technology nodes. Though Fig. 10c can be easily extendable for future technology nodes, the tunneling rate is insufficient due to non-availability of vertical tunneling mechanism. With the aforementioned features in device reliability, the study is extended to DC-performance comparison in terms of $I_{{\rm D}}$–$V_{{\rm G}}$ and $\hbox{SS}_{{\rm avg}}$, which is depicted in Fig. 11. The results signify that the proposed structure has 10-time improvement in $I_{{\rm on}}$ and 2-order advancement in $I_{{\rm on}}/I_{{\rm off}}$, and benefited with $\hbox{SS}_{{\rm avg}}$ (evaluated from $I_{{\rm off}}$ to $10^{-7}$ orders of current [38]) than the explored structure [7]. In summary, the proposed design has shown with improvements in DC than the recently reported devices and has further scope to improve through novel material options.

### Energy Efficient FATFETs

**Fig. 12:**
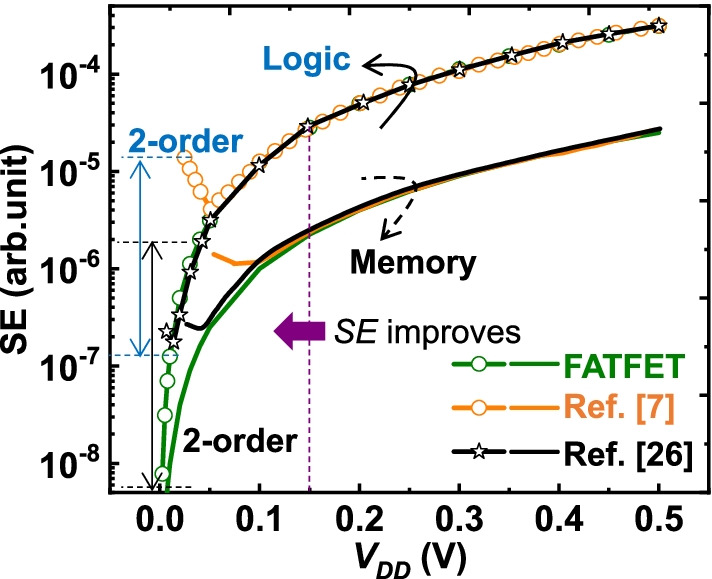
Comparison of SE variation with supply voltage scaling. It clears that the proposed structure (FATFET) reduces SE by 2-order (both in logic and memory environments) due to its high $I_{{\rm on}}{/}I_{{\rm off}}$ ratio

It is meaningful to perform energy efficiency tests on the proposed FATFETs being the TFETs as the energy efficient devices. The energy efficiency or switching energy (SE) is evaluated by [7]

27 $$\hbox{SE}\approx V_{{\rm DD}}^2(\xi +I_{{\rm off}}/I_{{\rm on}}),$$

and depicted in Fig. 12. Here, $V_{{\rm DD}}$ is the supply voltage, $\xi$ is the active time ratio with 1/800 and 1/10,000 for logic and memory applications. It is observed that the proposed (FATFET) has impressive performance in both logic and memory environments at supply voltages < 0.15 V. Specifically, 2-order reduction in SE is achieved in both logic and memory environments because of the impressive $I_{{\rm on}}{/}I_{{\rm off}}$ (Fig. 11).

## Conclusions

A promised TFET design called FATFET has been presented in this work. The modeling of ferroelectric parameters along with $V_{{\rm int}}$ calculations of MFIS geometry having QS and NQS has been addressed for the promised design. The benefits of ferroelectric with the design of FATFET has been shown in comparison to ATFET (without ferroelectric). Furthermore, the significance of $P_{{\rm r}}$ and $E_{{\rm c}}$ on FATFET for an optimized design is addressed. The results signifies that the high $E_{{\rm c}}$ and $P_{{\rm r}}$ are undesirable for stable performance of TFETs. With the optimized option of $P_{{\rm r}}$ and $E_{{\rm c}}$; FATFET has shown in delivering high $I_{{\rm on}}$, lower $I_{{\rm off}}$, low $\hbox{SS}_{{\rm avg}}$, and higher frequency terms ($f_{{\rm t}}$ and $f_{{\rm max}}$), respectively. Furthermore, the linearity analysis signifies that FATFET has the stabilized linear behavior (within the operating regime) compared to ATFET. Compared to recently reported structures, the proposed FATFET design proves to be impressive in terms of DC and SE.

## Data Availability

All data generated or analyzed during this study are included in this published article.

## Abbreviations

TFET: Tunneling field-effect transistor
LTFET: Line TFET
ATFET: Area TFET
FATFET: Ferroelectric ATFET
MFIS: Metal-ferroelectric-insulator
SS: Subthreshold swing
SE: Switching energy
HZO: Hafnium-zirconium-oxide
GAA: Gate-all-around
TCAD: Technology computer aided design
BTBT: Band-to-band tunneling
TAT: Trap-assisted-tunneling
L–K: Landau–Khalatnikov
QS: Quasi-static
NQS: Non-quasi-static
NC: Negative capacitance
Si: Silicon
SiGe: Silicon–germanium
NBE: Neutral beam etching
MOCVD: Metalorganic vapour-phase epitaxy
ALD: Atomic layer deposition
PLD: Pulsed layer deposition
EOT: Effective oxide thickness
TGF: Transconductance generation factor
